# Determinants of positive cervical cancer screening among reproductive‐age women in South Wollo Zone, Northeast Ethiopia

**DOI:** 10.1002/hsr2.527

**Published:** 2022-03-03

**Authors:** Abdulkerim Mengistu, Niguss Cherie, Elsabeth Addisu

**Affiliations:** ^1^ Department of Public Health Amhara Regional Health Bureau Bahir Dar Ethiopia; ^2^ Reproductive and Family Health Department School of Public Health, College of Medicine and Health Sciences, Wollo University Dessie Ethiopia

**Keywords:** case‐control, Ethiopia, positive cervical cancer screening, South Wollo Zone

## Abstract

**Background:**

Cervical cancer is one of the reproductive organ cancers found in women which commonly arises from the cervix. It is the second most prevalent cancer among women in developing countries including Ethiopia. However, the association between positive cervical cancer screening and modifiable behavioral risk has not been well characterized in developing countries.

**Objective:**

To identify determinants of positive cervical cancer screening among reproductive‐age women in the South Wollo Zone, Amhara region, northeast Ethiopia.

**Method:**

An unmatched case‐control study design was conducted from January 28 to April 12, 2020 in the South Wollo Zone. Four hundred ten clients participated in the study with 82 cases 328 controls. Study subjects were selected by systematic random sampling. Data entered using Epi data version 3.1 and analyzed using SPSS version 24. A bivariable and multivariable logistic regression model was done. The adjusted odds ratio with its 95% confidence interval (CI) was used to measure the strength and direction of the association and *P*‐value <.05 was declared as significant.

**Results:**

A total of 410 study subjects have participated with a 100% response rate. The mean age of respondents was found to be 35.58 (±8.05) years. Study participants having a history of sexually transmitted infections (adjusted odds ratio [AOR] = 3.69, 95% CI [1.70‐8.01]), having poor knowledge about cervical cancer (AOR = 2.31, 95% CI [1.32‐4.02]) and two or more lifetime sexual partners of women and husbands (AOR = 2.80, 2.55, 95% CI [1.22‐6.44, 1.28‐5.06]) respectively were independent predictors of positive cervical cancer screening.

**Conclusion and recommendation:**

Risk factors that determine positive cervical cancer screening were identified. Comprehensive strategies that are focused on addressing sexual behavior and knowledge gaps should be designed. Efforts on improving and cultivating those significant factors should be done by stakeholders to prevent cervical cancer.

**Abbreviations**
AOR: adjusted odds ratio
CI: confidence intervals
COR: crude odds ratio
FGAE: Family Guidance Association Ethiopia
HIV: human immune deficiency virus
HPV: humane papilloma virus
SPSS: statistical package for social sciences
STI: sexually transmitted infection

## 1 INTRODUCTION

Cervical cancer is one of the reproductive organ cancers found in women which commonly arises from the cervix.^1^ It is mainly caused by Human Papilloma Virus (HPV).^2^, ^3^, ^4^ Cervical cancer is preventable in most cases and curable if identified and treated in its precancerous stage.^5^ Cervical cancer is the second most prevalent cancer among women in developing countries including Ethiopia, and the largest killer cancer among women in those countries.^6^ Worldwide, a population of 2784 million women aged 15 years and older are at risk of developing cervical cancer.^7^ In Africa, according to the most recent estimates, 80 400 women are diagnosed with cervical cancer every year, the second most frequent cancer. 50 300 die from the disease every year. Rates vary substantially across regions, with the incidence and death rates in East Africa,^7^, ^8^ the region of Ethiopia belongs to, and West Africa five times as high as the rates in North Africa.^8^

In Ethiopia 29.43 million women ages 15 years and older are at risk of developing cervical cancer.^10^ Current estimates showed that 7095 women were newly diagnosed with cervical cancer and 4732 deaths resulting from it occur every year.^9^ Cervical cancer ranks as the second most frequent cancer among women in Ethiopia and between 15 and 44 years of age with an age‐standardized incidence rate of 26.4 per hundred thousand.^7^, ^9^ Cervical cancer causes the highest mortality rate compared to other types of cancers among women in Ethiopia.^10^ Cervical cancer has exerted negative consequences on health, economic, and social conditions.

The national guideline for cervical cancer prevention and control in Ethiopia showed that the majority of cancers (over 80%) in developing countries especially sub‐Saharan Africa are detected at a late stage.^11^ Nowadays HPV vaccine started in Ethiopia among 9 to 14 years school children starting from 2018, unfortunately still morbidity and mortality due to cervical cancer are high. This is attributable to low knowledge and generally poor health‐seeking behavior.^11^, ^12^ Evidence indicates that a significant part of the burden of cervical cancer is potentially prevented by early screening and treatment or by reducing and eliminating the risk factors.^13^, ^14^ Therefore, the “Ethiopian health sector transformation plan includes the national strategies of cervical cancer prevention and control programs to improve the health status of women.”^5^ However, the association between positive cervical cancer screening and modifiable behavioral risk has not been well characterized in the South Wollo Zone, Northeast Ethiopia. Therefore, this study was conducted to explore determinants of positive pre‐cervical lesion among women found in the South Wollo Zone. The finding of this study will help to plan appropriate intervention at all levels in the study area to improve the gap in cervical cancer prevention and control programs. Moreover, it will be used by other researchers as a baseline in future study.

## 2 METHODS AND MATERIALS

### 2.1 Study design, area and period

A facility‐based unmatched case‐control study was conducted in the South Wollo Zone from January 28, 2020 to April 12, 2020. The zone has a total of 10 primary hospitals, 129 health centers, 523 health posts, 134 private primary clinics, 47 medium clinics, and 1 non‐governmental health facility which gives preventive and curative services to the people. Twenty‐one health facilities provide cervical cancer screening and treatment services, which are 7 primary hospitals, one Family Guidance Association Ethiopia (FGAE) clinic, and 13 health centers.

### 2.2 Population and eligibility criteria

The source populations of cases were all women aged from 21 to 65 years with positive Pap smear results in the South Wollo Zone. The source populations for controls were all women aged from 21 to 65 years, who had Pap smear‐negative results. Women who had VIA positive test results for cases and VIA negative test results for controls in the randomly selected cervical cancer screening and treatment health facilities were study populations of this study. Severely ill clients not able to give responses were excluded from the study.

### 2.3 Sample size determination and procedure

The sample size was determined using Epi‐info version 7.1 by considering the assumptions proportion of women with a negative result from VIA who were exposed to multiple sexual partners as 30.57%, 95% CI, 80% power of the test with an adjusted odds ratio (AOR) of 2.17 and a 1:4 ratio of cases to controls shown in other studies.^15^ A total sample size of 410 was determined, including 82 cases and 328 controls, which also accounted for 10% non‐response. Among 21 health facilities, 6 health facilities were randomly selected using the lottery method and the total sample size was allocated to each selected health facility using proportional allocation according to average monthly client flow. The study subjects were selected using systematic random sampling from health facility flow.

### 2.4 Data collection procedures

Data were collected using pretested structured questionnaire through face‐to‐face interviews administered; in‐person by 10 trained BSC midwifery or nurses at the health facility level and the daily collected data were checked by trained supervisors. Questionnaires included socio‐demographic characteristics, reproductive health characteristics, lifestyle, sexual behavior characteristics, and knowledge of study subjects about cervical cancer.^15^, ^16^, ^18^, ^20^

### 2.5 Data quality assurance

The questionnaire was prepared in English and translated into Amharic. It was checked for consistency by back‐translation to English. A pre‐test was done and the data collection process was strictly supervised and data were checked for consistency and completeness daily. Incomplete and unclear questionnaires were returned to interviewers to be completed. The internal consistency of the knowledge questionnaire was checked using Cronbach's alpha value which was 0.88. Finally, the entered data were checked for completeness at the beginning, middle, and the last stage of the work and data cleaning was done at the end of the data entry.

### 2.6 Data processing and analysis

Data were entered into Epi data version 3.1 and then exported to SPSS version 24 for analysis. Data cleaning was done by running frequencies, cross‐tabulation, and sorting among reported cases or variables. The knowledge status of respondents about cervical cancer was identified by doing a composite analysis. A binary logistic regression analysis was done to describe the association between independent and dependent variables and a multivariable logistic regression analysis was used to show factors determining outcome variables. Variables that had a *P*‐value of .25 or less in the binary logistic regression were included in the multivariable logistic regression. Finally, the adjusted odds ratio with its 95% confidence interval (CI) with *P*‐value <.05 was considered statistically significant for all independent variables at the multivariable logistic regression. Before the final model Multicollinearity test was checked using variance inflation factors (VIF), to see the correlation between independent variables but, no collinearity was detected. The goodness of the model was checked by Hosmer‐Lemeshow's test statistic which was not significant.

### 2.7 Operational definitions

Case: A case was a woman aged from 21 to 65 years with positive for Visual Inspection of Acetic Acid (VIA) finding within 3 months before and during the actual data collection period.^31^

Control: A control was a woman aged 21 to 65 years with negative VIA finding during the actual data collection period.^31^

Knowledge: Out of 12 knowledge‐related questions, the respondents who respond sample mean score and above were classified as having “good Knowledge,” while those who responded below the sample mean score (0.01) were classified as having “poor Knowledge.”^29^

Multiple sexual partners: Women who have experienced two or more sexual partners in their lifetime.

Contraceptive history: Women who had the experience of any type of contraceptive use.

The user of contraceptive pills: Women who use oral contraceptive pills for family planning.

## 3 RESULTS

### 3.1 Socio‐demographic characteristics

All case and control women expected were interviewed which yields a 100% response rate. The mean age of cases and controls was found to be 35.58 (±8.05) and 35.8 (±8.5) years respectively. Among the study subjects, 34 (41.5%) of cases and 125 (38.1%) of controls were aged between 30 to 39 years (Table 1).

**TABLE 1:** Socio‐demographic characteristics of the study subjects in South Wollo Zone, Amhara region, northeast Ethiopia (n = 410)

Variables	Cases (%)	Control (%)
Educational status		
Non formal education	39 (47.6)	129 (39.3)
Primary education	20 (24.4)	74 (22.6)
Secondary and above	23 (28)	125 (38.1)
Religious status respondent		
Orthodox	40 (48.8)	158 (48.2)
Muslim	42 (51.2)	170 (51.8)
Marital status		
Single	18 (22)	57 (17.4)
Married	45 (54.9)	213 (64.9)
Others^a^	19 (23.2)	57 (17.4)
Occupational status		
House wife	55 (67)	204 (62.2)
Governmental employee	11 (13.4)	55 (16.8)
Others^b^	16 (19.5)	69 (21)
Age of respondents		
21‐29 y	21 (25.6)	84 (25.6)
30‐39 y	34 (41.5)	125 (38.1)
40‐49 y	20 (24.4)	95 (29)
≥50 y	7 (8.5)	24 (7.3)

^a^ Widowed and divorced.

^b^ Merchants, daily laborers and other self‐business.

### 3.2 Reproductive health‐related characteristics

Of all respondents, 62 (75%) of cases and 233 (71%) of controls had been ever used contraceptives. Regarding the duration of contraceptive use, 58.5% of cases and 57.6% of controls had the experience of contraceptive use for less than 5 years. Among the respondents, 17 (20.7%) of cases and 46 (14%) of controls had an experience of post‐coital bleeding last 1 year. Of all respondents, 70 (85.4%) of cases and 305 (93%) of controls had no family history of cervical cancer (Table 2).

**TABLE 2:** Reproductive health characteristics of the study subjects in the South Wollo Zone, Amhara region, northeast Ethiopia (n = 410)

Variables	Cervical cancer	
	Case (%)	Control (%)
Ever use contraceptives		
Yes	62 (75.6)	233 (71)
No	20 (24.4)	95 (29)
Type of contraceptive methods		
Oral contraceptive	12 (19.4)	39 (16.7)
Injectable	36 (58)	132 (56.7)
Others^a^	14 (22.6)	62 (26.6)
Duration of contraceptive use (y)		
<5 y	48 (77.4)	189 (81.1)
≥5 y	14 (22.6)	44 (18.9)
Menstrual history		
Regular	23 (28)	126 (38.4)
Sometimes irregular	26 (31.7)	72 (22)
Always irregular	22 (26.8)	83 (25.3)
No menses	11 (13.4)	47 (14.3)
Experience of post‐coital bleeding		
Yes	17 (20.7)	46 (14)
No	65 (79.3)	282 (86)
History of abortions		
Yes	32 (39)	77 (23.5)
No	50 (61)	251 (76.5)
Family history of cervical cancer		
Yes	12 (14.6)	23 (7)
No	70 (85.4)	305 (93)
History of ever pelvic infections		
Yes	24 (29.3)	57 (17.4)
No	58 (70.7)	271 (82.6)
History of genital ulcer or swelling		
Yes	24 (29.3)	48 (14.6)
No	58 (70.7)	280 (85.4)
Partner history of genital ulcer or swelling		
Yes	19 (23.2)	48 (14.6)
No	63 (76.8)	280 (85.4)

^a^ Implants and Intra utrine contraceptive device (IUCD).

### 3.3 Lifestyle and sexual behavior related characteristics

Among respondents, 17 (20.7%) of cases and 41 (12.5%) of controls were previously screened for cervical cancer in their lifetime (Table 3). Fifty‐three (64.6%) of cases and 270 (66.2%) of controls had never used condoms in their lifetime. The mean age at first marriage for cases and controls was 18.54 (±2.86) and 18.67(±3) years respectively. Regarding the history of sexually transmitted infection 19 (23.2%) of cases and 19 (5.8%) of controls had a history of STIs. Out of the participants, 20 (24.4%) of cases and 192 (58.5%) of controls had a history of two or more lifetime other sexual partners of husbands (Table 3).

**TABLE 3:** Lifestyle and sexual behavior characteristics of study subjects in the South Wollo Zone, Amhara region, northeast Ethiopia (n = 410)

Variables	Cases (%)	Control (%)
Ever smoke cigarette		
Yes	12 (14.6)	27 (8.2)
No	70 (85.4)	301 (91.8)
Ever used condom		
Always used	14 (17.1)	55 (16.8)
Sometimes used	15 (18.3)	56 (17.1)
Not used condom	53 (64.6)	217 (66.2)
History of sexually transmitted infections		
Yes	19 (23.2)	19 (5.8)
No	63 (76.8)	309 (94.2)
Partner history of sexually transmitted infection		
Yes	19 (23.2)	26 (7.9)
No	63 (76.8)	302 (92.1)
Previously tested for human immune deficiency virus (HIV)		
Yes	68 (82.9)	249 (75.9)
No	14 (17.1)	79 (24.1)
HIV status		
Positive	13 (19.1)	29 (11.6)
Negative	55 (80.9)	220 (88.4)
Early sexual initiation		
<15 y	18 (22)	88 (26.8)
15‐17 y	28 (34.1)	79 (24.1)
≥18 y	36 (43.9)	161 (49)
Lifetime multiple sexual partners		
No	12 (14.6)	153 (46.6)
One	20 (24.4)	76 (23.2)
Two or more	50 (61)	99 (30.2)
Lifetime other sexual partners of husbands		
One	62 (75.6)	136 (41.5)
Two or more	20 (24.4)	192 (58.5)

### 3.4 Knowledge status

Twenty‐two (31.7%) of cases and 171 (52.1%) of controls had good knowledge which had a sample mean score and above, but 56 (68.3%) of cases and 157 (47.9%) of controls had poor knowledge about cervical cancer which had to have below the sample mean score.

### 3.5 Determinants of positive cervical cancer screening

Those are educational status, marital status, early marriage, early childbirth, early sexual initiation, menstrual history, the experience of post‐coital bleeding, history of abortion, family history of cervical cancer, previously screened for cervical cancer and the results of the last cervical cancer screen, ever smoke cigarette, history of ever pelvic infection, history of sexually transmitted infections, partner history of sexually transmitted infections, history of genital ulcer or swelling, partner history of genital ulcer or swelling, ever tested for HIV, HIV status, multiple sexual partners, lifetime sexual partners of husbands and knowledge of women about positive cervical cancer screening were tested in bivariable binary logistic regression analysis with *P*‐value less than .25 and then imported to multivariable binary logistic regression.

Model fitness was checked by Hosmer and Lemeshow which was *P*‐value .3. Adjusting for other variables, The odds of having positive cervical cancer screening for women having a history of sexually transmitted infections were 3.69 times with (AOR = 3.69, 95% CI [1.70‐8.01]) more likely as compared to those who did not have a history of STI. Those women who had two or more lifetime sexual partners were 2.80 times with (AOR = 2.80, 95% CI [1.22‐6.44]) more likely to have positive cervical cancer screening than those who did not have two or more lifetime sexual partners. And the odds of having positive cervical cancer screening for women whose husbands had two or more lifetime sexual partners were 2.55 times with (AOR = 2.55, 95% CI [1.28‐5.06]) more likely as compared to those women whose husbands had no two or more lifetime sexual partners. Women who had poor knowledge about cervical cancer were 2.31 times with (AOR = 2.31, 95% CI [1.32‐4.02]) more likely to have positive cervical cancer screening than those who had good knowledge about cervical cancer (Table 4).

**TABLE 4:** Bivariable and multivariable binary logistic regression analysis on determinants of positive cervical cancer screening among women in the South Wollo Zone, Amhara region, Ethiopia (n = 410)

Variables	Case (n = 82)	Control (n = 328)	COR (95% CI)	AOR (95% CI)
Family history of cervical cancer				
Yes	12	23	2.27 (1.08, 4.79)*	1.71 (0.71, 4.08)
No	70	305	1.00	1.00
Previously screened for cervical cancer				
Yes	17	41	1.00	1.00
No	65	287	1.83 (0.98, 3.42)	1.85 (0.93, 3.69)
History of sexually transmitted infections				
Yes	19	19	4.91 (2.46, 9.79)*	3.69 (1.70, 8.0) **
No	63	309	1.00	1.00
Partner's history of sexually transmitted infections.				
Yes	19	26	3.50 (1.83, 6.72)*	1.11 (0.34, 3.64)
No	63	302	1.00	1.00
Lifetime multiple sexual partners				
No	12	153	1.00	1.00
One	20	76	3.36 (1.56, 7.22)*	2.33 (0.99, 5.48)
Two or more	50	99	6.44 (3.27, 12.69) *	2.80 (1.22, 6.44) *
Lifetime sexual partners of husbands				
One	20	192	1.00	1.00
Two or more	62	136	4.38 (2.53, 7.58)*	2.55 (1.28, 5.06) **
Knowledge of women about cervical cancer.				
Good knowledge	26	171	1.00	1.00
Poor knowledge	56	157	2.35 (1.40, 3.92)*	2.31 (1.32, 4.02) **

*Note*: *P*‐value: *Significant (*P* < .05); **highly significant (*P* < .01), 1 (reference category).

## 4 DISCUSSION

The aim of this study was to explore determinant factors of positive cervical screening among women found in the South Wollo Zone. Adjusting for other variables, study participants who had a history of sexually transmitted infections, history of lifetime multiple sexual partners, husband's history of lifetime multiple sexual partners and poor knowledge about cervical cancer were identified as predictors of positive cervical cancer screening.

Women who had a history of sexually transmitted infections were 3.69 times more likely to be positive cervical cancer screening as compared to those women who did not have a history of sexually transmitted infections. This finding is similar to a study done in Addis Abeba, Adama town in Ethiopia and Nairobi Kenya, Zimbabwe.^23^, ^30^ But it is different from the study done in Yergalem General hospital southern Ethiopia those where women who had a history of STI were less likely to have positive cervical cancer screening than this study finding.^31^ This might be due to sampling size, study design, socio‐cultural and population characteristics differences. The implication of this finding might be due to a lack of comprehensive and complemented service provision on STI and cervical cancer prevention and control.

Women who had two or more lifetime sexual partners were 2.80 times more likely to have positive cervical cancer screening than those who did not have two or more lifetime sexual partners. This showed that an increase in the number of sexual partners has raised the risk of developing cervical cancer. This finding is consistent with the study conducted in Addis Abeba, Adama town and Tigray region, Ethiopia.^23^ But it is different from the study done in Yergalem General Hospital in Ethiopia those where women who had two or more lifetime sexual partners were less likely to have positive cervical cancer screening than this study finding.^31^ This might be due to study design, sociocultural and sample size differences. Moreover, having multiple sexual partners increases the risk of infection with high‐risk human papillomavirus (HPV).^32^ In this study, women who had a history of lifetime multiple sexual partners were positively associated with positive cervical cancer screening. However, in the study conducted in North Central Nigeria, those who had two or more lifetime sexual partners were negatively associated with the risk of developing cervical cancer.^31^ This might be due to socio‐demographic, cultural, and study design differences. The implication of this finding might be due to improper implementation of behavioral change communication strategies to change or limit lifetime multiple sexual practices among women. And also, women might not understand the impact of multiple sexual partners as a risk factor of cervical cancer.

Women whose husbands had two or more lifetime sexual partners were 2.55 times more likely to develop Positive cervical cancer screening than those women whose husbands had no two or more lifetime sexual partners. This finding is consistent with the study done in Algeria, Addis Abeba and Jimma university specialized hospital southwest Ethiopia,.^23^ Furthermore, early sexual activity and multiple sexual partners are cofactors that are independently associated with abnormal cytology and cervical cancer.^34^ This is higher because those women have a higher risk of acquiring HPV infection, which is the causative agent for cervical cancer.^35^ The implication of this finding might be due to improper implementation of behavioral change communication strategies to change or limit lifetime multiple sexual practices among husbands. And also, husbands might not understand the impact of multiple sexual partners as a risk factor of cervical cancer. This needs strong intervention to improve risky sexual behaviors among youths School‐based education can be a solution for boys and girls at younger ages at youth centers to reach out to school youths.

Women who had poor knowledge about cervical cancer were 2.31 times more likely to have positive cervical cancer screening than those who had good knowledge about cervical cancer. This finding is similar to the study done in the Jimma town, Tigray region Ethiopia and Cambodia.^26^, ^29^, ^36^ But it is different from the study done in Hossana town in Ethiopia those where women who had poor knowledge about cervical cancer were less likely to develop cervical cancer than this study finding.^35^ The possible reason might be the study design, socio‐cultural, and sample size differences. On the other hand, the study done in Nigeria showed that knowledge about cervical cancer and screening was extremely poor and the overwhelming majority of the respondents could not correctly identify the risk factors, symptoms and means of prevention of cervical cancer and more than 95% of respondent had very poor knowledge of cervical cancer and screening.^36^ This finding was also, supported by the national guideline for cervical cancer prevention and control in Ethiopia showed that the majority of cancers (over 80%) in sub‐Saharan Africa are detected at a late stage, predominantly due to lack of information about cervical cancer and death of prevention services.^19^ Therefore, it is one of the important predictors of the risk of developing cervical cancer. The implication of this finding might be due to lack of awareness, lack of information and improper giving of health education about cervical cancer.

## 5 LIMITATION OF THE STUDY

Unlike a population‐based study design, this study recruited research participants in healthcare facilities. So, even with a very high response rate, the sample recruited only represented those who attended healthcare facilities in South Wollo Zone. Whether this pattern also applied to those who did not attend any healthcare facilities remains unknown. Social desirability and recall bias may be introduced in the time of data collection. Anonymity was kept to minimize social desirability bias.

## 6 CONCLUSION

This study identified several factors correlated with women having pre‐cervical cancer lesions in Ethiopia. It may be helpful if the Government of Ethiopia develops comprehensive and complimented strategies to prevent and control STIs at all levels, planning interventions focusing on behavioral changes, encouraging women to delay sexual debut, school‐based sexuality education, and clinical screening for women having sexually transmitted infection.

## FUNDING

The research got a grant from Amhara Region Health Bureau However; the granting agency has no role in the design of the study and collection, analysis, and interpretation of data, and in writing the manuscript.

## CONFLICT OF INTEREST

The authors declare that they have no competing interests.

## AUTHOR CONTRIBUTIONS

Conceptualization: Niguss Chere, Abdulkerim Mengistu, Elsabeth Addissu

Formal analysis: Niguss Chere, Abdulkerim Mengistu, Elsabeth Addissu

Investigation: Abdulkerim Mengistu, Elsabeth Addissu, Elsabeth Addissu

Methodology: Niguss Chere, Abdulkerim Mengistu, Elsabeth Addissu

Project administration: Abdulkerim Mengistu

Software: Abdulkerim Mengistu, Elsabeth Addissu

Supervision: Niguss Chere

Validation: Niguss Chere

Writing – original draft: Niguss Chere, Abdulkerim Mengistu, Elsabeth Addissu

Writing – review & editing: Niguss Chere, Elsabeth Addissu

All authors read and approved the final manuscript and have an equal contribution.

AM‐initiation of the study, design, analysis, and writing of the manuscript, NC and EA‐ assisted in the design, participated in organizing the data collection process, analysis, and report writing, and writing the manuscript. All authors read and approved the final manuscript and have an equal contribution.

## TRANSPARENCY STATEMENT

The lead author (Niguss Cherie) affirms that this manuscript is an honest, accurate, and transparent account of the study being reported; that no important aspects of the study have been omitted; and that any discrepancies from the study as planned have been explained.

## ETHICS STATEMENT

The study protocol was approved by the Institutional Research Review Board of Wollo University College of Health Sciences and Community Service Ethical Review Committee. Written Permission was obtained from all relevant authorities in the South Wollo Zone health department as well as participating health facilities. Then the participants of the study were informed about the aim and purpose of the study, the importance of their participation, and their rights, and informed consent was obtained from the study subjects. Participation in the study was voluntary and participants were informed of the right to withdraw from the study. Data collection was conducted confidentially.

## Data Availability

The data supporting the findings of this study are available from the corresponding author upon request. The corresponding author had full access to all of the data in this study and takes complete responsibility for the integrity of the data and the accuracy of the data analysis.
